# Reply to: Is there a kindling effect in COPD exacerbations?

**DOI:** 10.1183/13993003.02055-2024

**Published:** 2024-12-12

**Authors:** David M.G. Halpin, Heath Heatley, David Price

**Affiliations:** 1 University of Exeter Medical School, College of Medicine and Health, University of Exeter, Exeter, UK; 2 Observational and Pragmatic Research Institute, Singapore; 3 Optimum Patient Care, Aylsham, UK; 4 Centre of Academic Primary Care, Division of Applied Health Sciences, University of Aberdeen, Aberdeen, UK

## Abstract

We thank A.I. Papaioannou and K. Bartziokas for their interest in our study of the relationship between exacerbation history and blood eosinophil count prior to a diagnosis of COPD and the risk of subsequent exacerbations [1]. As they point out, it is well known that in patients with an established diagnosis and on maintenance treatment, the prior exacerbation history is the best predictor of future exacerbation risk, but our study was the first to show that in newly diagnosed patients, a single exacerbation prior to diagnosis of COPD is associated with a significant risk of exacerbations over the next 12 months and more frequent or severe exacerbations prior to diagnosis are associated with a higher risk.

## Shareable abstract

**Real world studies have shown that over half of the patients having an exacerbation will not have one in the next year, suggesting that the majority of patients are not subject to a “kindling effect”** https://bit.ly/3O016tT

*Reply to A.I. Papaioannou and K. Bartziokas:*

We thank A.I. Papaioannou and K. Bartziokas for their interest in our study of the relationship between exacerbation history and blood eosinophil count prior to a diagnosis of COPD and the risk of subsequent exacerbations [1]. As they point out, it is well known that in patients with an established diagnosis and on maintenance treatment, the prior exacerbation history is the best predictor of future exacerbation risk, but our study was the first to show that in newly diagnosed patients, a single exacerbation prior to diagnosis of COPD is associated with a significant risk of exacerbations over the next 12 months and more frequent or severe exacerbations prior to diagnosis are associated with a higher risk. A.I. Papaioannou and K. Bartziokas question whether this represents what they call a “kindling effect”, whereby previous exacerbations lead to some sort of “sensitisation” that lowers the threshold for triggers of subsequent events, leading to an ever increasing the frequency and severity of exacerbations.

COPD exacerbations are mainly triggered by external factors such as viral or bacterial infections or air pollution, but some are characterised by high airway eosinophil count [2] and there is evidence that host factors, including the lung microbiome, influence the type of exacerbation [3, 4]. Viral exacerbations appear to occur at random but bacterial and eosinophilic exacerbations appear more likely to occur repeatedly in the same individual [4]. This might support the concept that patients have an inflammatory endotype that predisposes them to specific types of exacerbation. Alternatively, it might suggest that previous exacerbations destabilise the lung microbiome, thereby increasing the risk of future exacerbations. However, although previous exacerbations are the major risk factor for future exacerbations, real world studies have shown that more than half of the patients having an exacerbation will not have one in the next year and over half of patients having a further exacerbation in the next year will not have one the following year [5]. Moreover, approximately half of frequent exacerbators had either zero or only one exacerbation in the subsequent year [6]. This suggests that the majority of patients are not subject to a “kindling effect”.

## Shareable PDF

10.1183/13993003.02055-2024.Shareable1 This PDF extract can be shared freely online.
Shareable PDF ERJ-02055-2024.Shareable
