# Supplementary material for: Reply to: Is there a kindling effect in COPD exacerbations?
Source: Eur Respir J. 2024 Dec 12;64(6):2402055. doi: 10.1183/13993003.02055-2024 (PMC11635379; doi:10.1183/13993003.02055-2024)
Supplement: Supplementary file 1 [file ERJ-02055-2024.Shareable.pdf]

SHAREABLE PDF

Reply to: Is there a kindling effect in COPD exacerbations?

David M.G. Halpin [1,2], Heath Heatley [3] and David Price [2,3,4]

[1]University of Exeter Medical School, College of Medicine and Health, University of Exeter, Exeter, UK. [2]Observational and Pragmatic Research Institute, Singapore. [3]Optimum Patient Care, Aylsham, UK. [4]Centre of Academic Primary Care, Division of Applied Health Sciences, University of Aberdeen, Aberdeen, UK.

Corresponding author: David M.G. Halpin (d.m.g.halpin@ex.ac.uk)

Check for updates

Shareable abstract (@ERSpublications)
Real world studies have shown that over half of the patients having an exacerbation will not have one in the next year, suggesting that the majority of patients are not subject to a "kindling effect" https://bit.ly/3O016tT

Cite this article as: Halpin DMG, Heatley H, Price D. Reply to: Is there a kindling effect in COPD exacerbations?. *Eur Respir J* 2024; 64: 2402055 [DOI: 10.1183/13993003.02055-2024].

This extracted version can be shared freely online.

Copyright ©The authors 2024.

This version is distributed under the terms of the Creative Commons Attribution Licence 4.0.

Received: 15 Oct 2024
Accepted: 21 Oct 2024

Reply to A.I. Papaioannou and K. Bartziokas:

We thank A.I. Papaioannou and K. Bartziokas for their interest in our study of the relationship between exacerbation history and blood eosinophil count prior to a diagnosis of COPD and the risk of subsequent exacerbations [1]. As they point out, it is well known that in patients with an established diagnosis and on maintenance treatment, the prior exacerbation history is the best predictor of future exacerbation risk, but our study was the first to show that in newly diagnosed patients, a single exacerbation prior to diagnosis of COPD is associated with a significant risk of exacerbations over the next 12 months and more frequent or severe exacerbations prior to diagnosis are associated with a higher risk. A.I. Papaioannou and K. Bartziokas question whether this represents what they call a "kindling effect", whereby previous exacerbations lead to some sort of "sensitisation" that lowers the threshold for triggers of subsequent events, leading to an ever increasing the frequency and severity of exacerbations.
